# The impact of preload on 3-dimensional deformation parameters: principal strain, twist and torsion

**DOI:** 10.1186/s12947-017-0111-x

**Published:** 2017-09-12

**Authors:** Hyo-Suk Ahn, Yong-Kyun Kim, Ho Chul Song, Euy Jin Choi, Gee-Hee Kim, Jung Sun Cho, Sang-Hyun Ihm, Hee-Yeol Kim, Chan Seok Park, Ho-Joong Youn

**Affiliations:** 1 Divisions of Cardiology, College of Medicine, Catholic University of Korea, 222 Banpo-daero, Seocho-gu, Seoul, 06591 Republic of Korea; 2 Nephrology, College of Medicine, Catholic University of Korea, Seoul, South Korea

**Keywords:** Three-dimensional echocardiography, Myocardial strain, Hemodialysis

## Abstract

**Background:**

Strain analysis is feasible using three-dimensional (3D) echocardiography. This approach provides various parameters based on speckle tracking analysis from one full-volume image of the left ventricle; however, evidence for its volume independence is still lacking.

**Methods:**

Fifty-eight subjects who were examined by transthoracic echocardiography immediately before and after hemodialysis (HD) were enrolled. Real-time full-volume 3D echocardiographic images were acquired and analyzed using dedicated software. Two-dimensional (2D) longitudinal strain (LS) was also measured for comparison with 3D strain values.

**Results:**

Longitudinal (pre-HD: −24.57 ± 2.51, post-HD: −21.42 ± 2.15, *P* < 0.001); circumferential (pre-HD: −33.35 ± 3.50, post-HD: −30.90 ± 3.22, *P* < 0.001); and radial strain (pre-HD: 46.47 ± 4.27, post-HD: 42.90 ± 3.61, P < 0.001) values were significantly decreased after HD. The values of 3D principal strain (PS), a unique parameter of 3D images, were affected by acute preload changes (pre-HD: −38.10 ± 3.71, post-HD: −35.33 ± 3.22, *P* < 0.001). Twist and torsion values were decreased after HD (pre-HD: 17.69 ± 7.80, post-HD: 13.34 ± 6.92, *P* < 0.001; and pre-HD: 2.04 ± 0.86, post-HD:1.59 ± 0.80, respectively, P < 0.001). The 2D LS values correlated with the 3D LS and PS values.

**Conclusion:**

Various parameters representing left ventricular mechanics were easily acquired from 3D echocardiographic images; however, like conventional parameters, they were affected by acute preload changes. Therefore, strain values from 3D echocardiography should be interpreted with caution while considering the preload conditions of the patients.

## Background

Two-dimensional (2D) echocardiography is the most widely used examination method for left ventricular (LV) dimension and function assessment, and the high frame rate of 2D speckle tracking echocardiography (STE) allows for the precise assessment of myocardial function through the analysis of myocardial deformation. Longitudinal, circumferential and radial movements, which represent dynamic LV changes during a cardiac cycle, can be measured from parasternal and apical echocardiographic windows. However, assumptions about LV geometry were inevitably the limitation of this method [1].

Real-time three-dimensional (3D) echocardiography is better correlated with cardiac MRI than 2D echocardiography in the measurement of LV volumes [2]. It is particularly useful for evaluating cardiac volumes in patients with cardiomyopathies, whose hearts possess more complex structures than the normal heart, and allows for a more accurate measurement of LV volume even in cases of geometrically asymmetric LV aneurysms [3, 4]. Moreover, 3D echocardiography also proved to be superior to 2D echocardiography when evaluating cardiac valves [5]. Studies other than volume measurements can also be performed; for example, Yodwut et al. showed the clinical efficacy of 3D echocardiography for the assessment of LV diastolic function [6].

Recent studies have shown that 3D strain measurements of the left ventricle using speckle tracking can represent LV mechanical function and can be achieved using several types of vendor-dependent and independent software. Strain assessed by 3D STE can predict the prognosis of patients who have suffered acute myocardial infarction and heart failure [7, 8]. It can be measured even at frame rates as low as 18 frames/sec [9], and the rotational motion of the left ventricle can also be reliably measured from 3D echocardiography [10].

Principal strain (PS) is a newly introduced parameter in cardiology that can be obtained from 3D echocardiography. This is accomplished by recognizing the direction along which strain occurs (the so-called principal direction) and the entities of actual deformation along the principal direction. It characterizes 3D strain properties, including longitudinal and circumferential strain values as well as torsional shear deformation, and can therefore represent dynamic 3D movements of the left ventricle [11, 12].

Longitudinal and circumferential strain calculated by 2D STE was demonstrated to be affected by acute preload changes caused by normal saline infusion [13]. However, there are little data on the effects of preload on the various parameters that can be acquired from 3D STE, such as strain, twist and torsion. Moreover, it is not known whether the newly introduced echocardiographic parameter of PS is volume independent. Although twist can be measured from 2D STE, it is greatly affected by the position of the parasternal images [14]. Therefore, 3D STE is anticipated to be a useful tool for a more accurate analysis of twist and torsion.

We hypothesized that various kinds of strain values from 3D STE, including PS, twist and torsion, may be influenced by acute volume change. We attempted to test this in a group of patients with end-stage renal disease (ESRD) who underwent periodic hemodialysis (HD) and experienced a subsequent preload reduction.

## Methods

### Patients

Patients who were regularly undergoing HD at Bucheon St. Mary’s Hospital in Bucheon, South Korea, were recruited. All subjects were enrolled on a prospective basis. Among the 98 patients who were regularly undergoing HD on a periodic basis for at least 1 month prior to enrollment in the institute, 63 patients volunteered. An experienced echocardiographer who was blinded to the study design performed screening echocardiography on all volunteer subjects. Before and after the screening echocardiography, the exclusion criteria were as follows: (i) current acute coronary syndrome; (ii) previous cardiac surgery or device implantation; (iii) current presence or previous history of significant arrhythmia, such as atrial fibrillation; (iv) LV ejection fraction less than 50%; (v) evidence of major valvular heart disease (i.e., any degree of mitral or aortic stenosis; more than a mild degree of mitral, aortic, or tricuspid regurgitation; and the presence of a prosthetic valve); and (vi) a poor echocardiographic window that was not appropriate for interpretation.

Five patients were excluded after the screening echocardiography due to significant valve dysfunction (*n* = 2), arrhythmia detected during echocardiographic examination (*n* = 1), LV regional wall motion abnormalities (*n* = 1) and a poor echocardiographic window (*n*= 1); therefore, 58 subjects were finally enrolled in this analysis.

### Echocardiographic examination

Transthoracic 2D and real-time 3D examinations were carried out by an experienced echocardiographer who was blinded to the study design while the patients were in the left lateral decubitus position.

A commercially available ultrasound machine (Vivid E9; General Electric Health Care, Milwaukee, WI) equipped with phased array transducers (M5S-D and 4 V–D) was applied for echocardiographic examination. Echocardiograms were performed immediately before and less than 30 min after a single dialysis session.

From the M-mode measurements, LV dimension and diastolic LV septal and posterior thickness were determined in the parasternal long-axis view. The 2D data were acquired from the parasternal long-axis and short-axis views and the three standard apical views. For each view, three consecutive cardiac cycles were recorded during quiet respiration. LV mass was determined using the area–length method and was corrected for body surface area. LV volume, ejection fraction and left atrial volume were determined using the modified Simpson’s method from apical 4- and 2-chamber views. Pulsed Doppler echocardiography of transmitral velocities was used to determine the peak E velocity, peak A velocity and the ratio between peak E and A velocities (E/A ratio). LV early diastolic e’ velocity and late diastolic a’ velocity were determined at the septal and lateral portion of the mitral annulus by Doppler tissue imaging and then averaged for evaluation. These measurements were obtained by setting the sample volume at the septal and lateral annulus and then recording at a sweep of 100 mm/s. All examinations were performed according to the recommendations of the American Society of Echocardiography and the European Association of Cardiovascular Imaging [15].

During 3D imaging, to achieve a high frame rate and the highest spatial resolution, the pyramidal scan volume was focused on the LV volume and the data sets were acquired during a single breath hold, taking care to include the whole left ventricle.

### 2D and 3D speckle tracking analysis

2D and 3D images were kept in a proprietary format (GE Healthcare, Milwaukee, WI) with Digital Imaging and Communications in Medicine Wrapper. Images were downloaded into a software package (EchoPAC version 12.0; GE Healthcare, Milwaukee, WI) and then exported into ImageArena Software (TomTec Imaging Systems; Unterschleissheim, Germany) for analysis, including 2D and 3D strain analyses. The 2D strain data were used to validate the 3D strain data. For this purpose, three apical B-mode sequences (2-, 3- and 4-chamber views) were recorded at an optimal frame rate (>30 frames/sec, also ensuring >30 frames/heartbeat) and optimal resolution for myocardium while focusing the image on the entire left ventricle; the images were kept in the DICOM format for post-processing. LS was assessed using the speckle tracking method at the endocardial level with Cardiac Performance Analysis software (Version 1.2, TomTec Imaging Systems; Unterschleissheim, Germany). Global values were then calculated as averages from the segments in each view.

Three-dimensional images were analyzed using commercially available vendor-independent software (4D LV analysis version 3.1; TomTec Imaging Systems; Unterschleissheim, Germany). First, the LV long axis was designated in the three apical views (four, three and two chamber) by the operator. The software distinguished the LV endocardial border and tracked it for an entire cardiac cycle. Last, the curves of PS strain were determined using the standard 16-segment model.

The reliability of the 3D measurements was estimated by comparing the 3D global longitudinal strain (LS) parameters with the corresponding parameter measured by 2D analysis from the relevant subjects. Correlations between 3D global PS and 2D global LS were also investigated. We also compared the differences in the 3D data analyzed by vendor-independent (4D LV analysis version 3.1; TomTec Imaging Systems, Unterschleissheim, Germany) and vendor-dependent (Echo PAC version 12.0; General Electric Health Care, Milwaukee, WI) software. Area strain (AS; i.e., area change ratio) could not be determined by vendor-independent software, and PS could not be calculated by vendor-dependent software. We directly compared only the values of 3D global longitudinal, circumferential, and radial strain that were acquired from the same 3D echocardiographic images using two different 3D image analysis systems. We also investigated the correlation between PS and AS.

### Reproducibility

To evaluate intra-observer variability in the offline analysis, 20 patients were randomly selected and analyzed by the same operator with at least a 1-week interval between the two analyses. To assess the effect of inter-observer variability, the same 20 subjects were analyzed in a random order at different times using the same software by a second investigator who was blinded to the results from the first investigator.

### Statistical analysis

All data analyses were performed using the statistical analysis software package R version 3.4.1 [16]. All continuous variables were shown as the mean ± standard deviation (SD). Differences in continuous variables between the pre- and post-HD states were estimated using the paired t-test. The x^2^ and Fisher’s exact tests were applied to assess differences between categorical variables. The correlations between 3D PS, 3D LS and 2D LS were evaluated by Pearson’s correlation coefficient. Linear regression analysis between the values of 2D and 3D strain was performed. The correlation between 3D PA and AS was also evaluated by Pearson’s correlation coefficient and linear regression analysis [17].

Reliability was evaluated using the intra-class correlation coefficient (ICC) to determine both intra- and inter-observer variability using an R package for the ICC [18]. The inter-software variability was determined by the ICC. The clinical significance of the ICC was interpreted as follows: excellent, ICC ≥ 0.80; good, 0.60 ≤ ICC < 0.80; moderate, 0.40 ≤ ICC < 0.60; and poor, ICC < 0.40. Bland-Altman analyses were also performed. The R package BlandAltmanLeh was used for this purpose [19]. *P*-values <0.05 were considered statistically significant.

## Results

3D STE analysis successfully performed in all 58 patients, but the measurement of LS from 2D STE could not be performed due to poor image quality in one patient pre-HD and two patients post-HD.

The clinical characteristics of the subjects are summarized in Table 1. The mean ultrafiltration rate was 11.5 ± 4.6 mL/kg/h. The mean age of the subjects was 59 ± 12 years, and 50% of the participants were male (*n* = 29). The most common cause of ESRD was diabetes mellitus (*n* = 36, 62%), the second was hypertension (*n* = 17, 29%), and the third was chronic glomerulonephritis (n = 3, 5%) (Table 1).

**Table 1:** Baseline characteristics of patients

	Total (*n* = 58)
Age (years)	59 ± 12
Male (%)	29 (50)
Ultrafiltration rate (mL/kg/h)	11.5 ± 4.6
Hemoglobin (g/dL)	11.4 ± 1.1
Hematocrit (%)	33.4 ± 5.2
Albumin (g/dL)	4.1 ± 0.3
Calcium × phosphate product	48.7 ± 17.5
Cause of renal failure (%)	
Diabetes mellitus	36 (62)
Hypertension	17 (29)
Chronic glomerulonephritis	3 (5)
Others causes or cryptogenic	2 (4)
Stroke (%)	6 (10)
Current medication (%)	
ACEi^a^	0 (0)
ARB^b^	42 (72)
CCB^c^	34 (62)
Beta blocker	37 (64)
Statin	10 (17)

^a^ *ACEi* angiotensin-converting enzyme inhibitor, ^b^ *ARB* angiotensin receptor blocker, ^c^ *CCB* calcium channel blocker

Table 2 presents the changes in blood pressure and heart rate after acute preload reduction caused by HD. Systolic, diastolic and mean blood pressure were significantly lowered after HD. The differences in heart rate based on HD status were statistically insignificant.

**Table 2:** Blood pressure and heart rate

	Pre-HD (*n* = 58)	Post-HD (*n* = 58)	*P value*
Blood pressure (mmHg)			
Systolic	155.4 ± 16.0	129.6 ± 19.4	<0.001
Diastolic	79.8 ± 13.1	74.4 ± 12.6	<0.001
Mean	105.0 ± 11.2	92.8 ± 13.1	<0.001
Heart rate (beats/min)	68.9 ± 10.1	70.6 ± 10.2	0.107

*HD* hemodialysis

Conventional echocardiographic parameters are summarized in Table 3. Many echocardiographic parameters that depict LV systolic and diastolic function were changed after HD. The end-diastolic, end-systolic and stroke volumes of the left ventricle were decreased after HD. LV ejection fraction was also altered by preload reduction. Diastolic parameters were evaluated by pulsed-wave Doppler, including the peak E wave velocity, E/A ratio, E wave deceleration time and isovolumetric relaxation time, which showed significantly different values based on HD status. Left atrial volume index, peak early diastolic tissue velocity (e’) and the ratio between peak early diastolic mitral inflow velocity and peak early diastolic tissue velocity (E/ e’) were also affected (Table 3).

**Table 3:** Conventional echocardiographic parameters

	Pre-HD (*n* = 58)	Post-HD (*n* = 58)	*P value*
Septal thickness (mm)	10.9 ± 1.7	10.7 ± 2.0	0.292
Posterior wall thickness (mm)	10.3 ± 2.1	10.4 ± 1.8	0.692
LV end-diastolic dimension (mm)	51.4. ± 5.5	48.1 ± 5.7	<0.001
LV end-systolic dimension (mm)	31.2 ± 4.2	29.3 ± 4.5	<0.001
LV end-diastolic volume (mL/ m^2^)	67.4 ± 15.7	56.2 ± 17.9	<0.001
LV end-systolic volume (mL/ m^2^)	23.6 ± 6.5	20.6 ± 7.8	<0.001
Stroke volume (mL/m^2^)	42.2 ± 10.0	35.7 ± 10.8	0.033
LVEF (%)	65.1 ± 4.4	63.6 ± 4.3	0.023
LV mass index (g/m^2^)	119.9 ± 29.4	109.9 ± 30.0	<0.001
E wave (cm/s)	80.3 ± 24.7	58.4 ± 23.7	<0.001
A wave (cm/s)	87.5 ± 19.8	85.6 ± 29.1	0.685
E/A ratio	0.95 ± 0.34	0.74 ± 0.30	<0.001
DT (ms)	209.2 ± 43.1	224.0 ± 46.2	0.015
IVRT (ms)	84.4 ± 15.6	101.4 ± 23.8	<0.001
A wave duration (ms)	147.6 ± 17.7	148.9 ± 18.4	0.640
LA volume index (mL/m^2^)	49.7 ± 13.8	36.9 ± 14.4	<0.001
e’ (cm/s)	6.61 ± 1.62	6.00 ± 1.50	0.001
a’ (cm/s)	9.31 ± 2.52	8.91 ± 1.42	0.298
E/e’	12.83 ± 5.08	10.05 ± 4.22	<0.001

*LV* left ventricle, *LVEF* Left ventricular ejection fraction, E wave: Peak early diastolic mitral inflow velocity, A wave: Peak late diastolic mitral inflow velocity, *DT* Deceleration time of peak early diastolic mitral inflow velocity, *IVRT* Isovolumic relaxation time, *LA* left atrium, e’: Average of peak early diastolic tissue velocity measured at septal and lateral mitral annulus, a’: Average of peak late diastolic tissue velocity measured at septal and lateral mitral annulus, E/e’: Ratio between peak early diastolic mitral inflow velocity and peak early diastolic tissue velocity

**Fig. 1:**
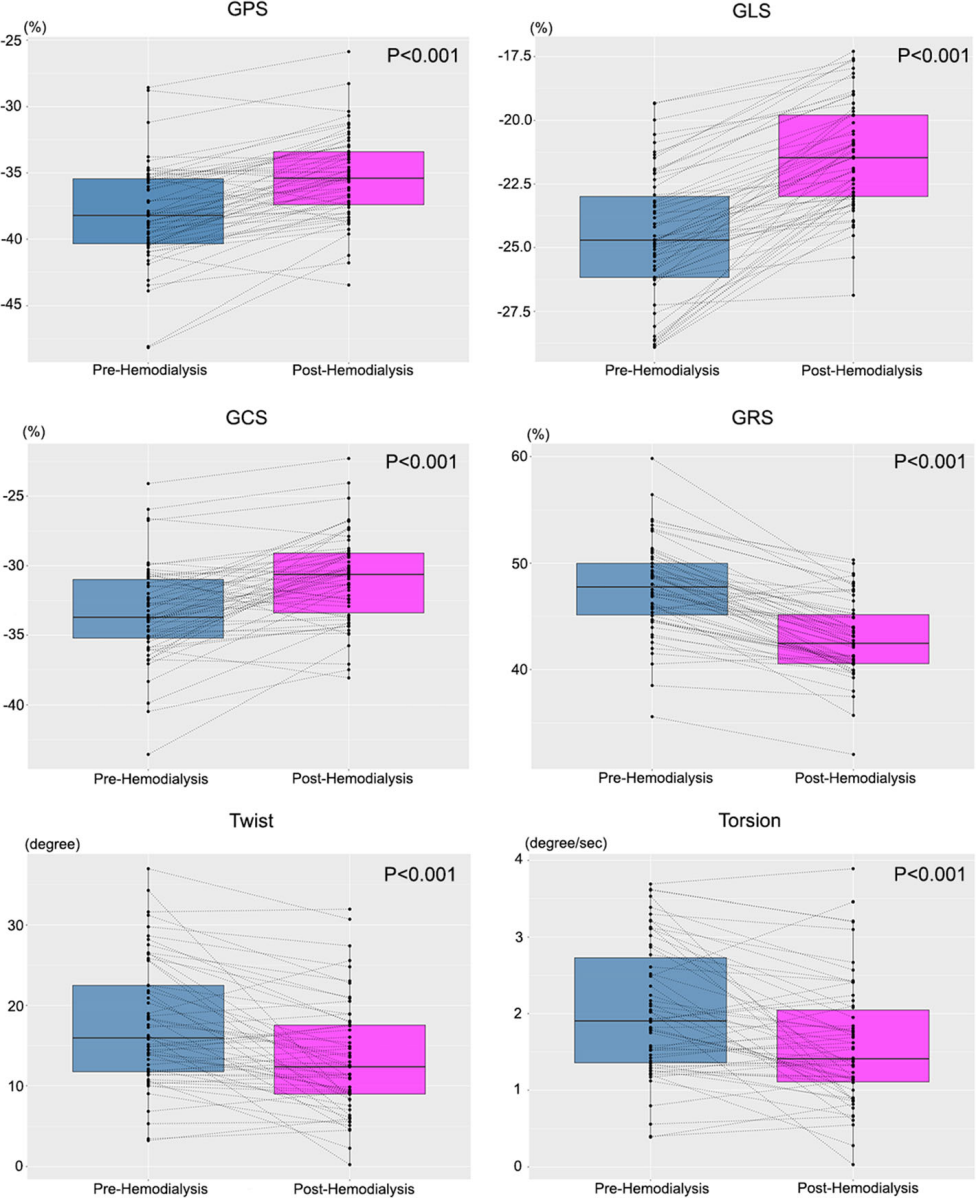
Global principal, longitudinal, circumferential and radial strain before and after hemodialysis. Twist and torsion according to hemodialysis status are also presented. GPS: global principal strain; GLS: global longitudinal strain; GCS: global circumferential strain; and GRS: global radial strain

**Fig. 2:**
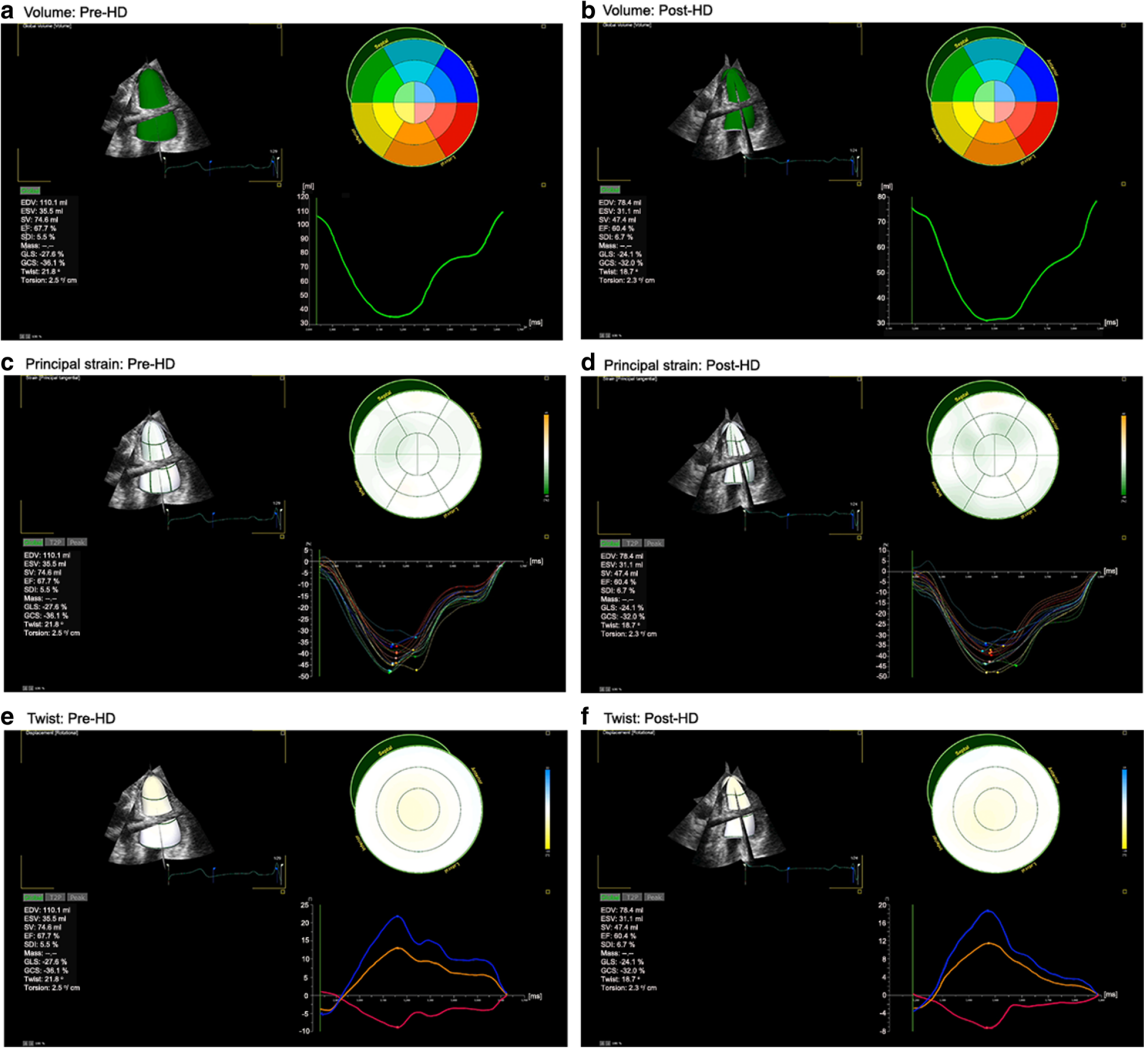
Comparison of the volume, principal strain and torsion of the left ventricle based on the acute preload reduction. Pre-hemodialysis (**a**, **c** and **e**); and post-hemodialysis (**b**, **d** and **f**).

Table 4 summarizes the LV volumes, strain, twist and torsion measured by 3D STE. The frame rates were not significantly different between pre- and post-HD patients. All parameters were easily obtained from one 3D LV full-volume image, and they were all affected by acute preload reduction. The novel parameter 3D PS was also changed after HD (pre-HD: −38.10 ± 3.71, post-HD: −35.33 ± 3.22, *P* < 0.001). The values of twist and torsion were decreased according to the preload change caused by HD (pre-HD: 17.69 ± 7.80 post-HD:13.34 ± 6.92, *p* < 0.001; pre-HD: 2.04 ± 0.86 post-HD: 1.59 ± 0.80, respectively, P < 0.001) (Table 4, Figs. 1 and 2).

**Table 4:** Left ventricular volumes, strains, twist, and torsion in patients with end-stage renal disease (ESRD) before and after hemodialysis (HD) measured by 3-dimensional speckle tracking echocardiography

	Pre-HD (*n* = 58)	Post-HD (*n* = 58)	*P value*
Frame rates (/min)	26.3 ± 4.6	25.2 ± 4.7	0.120
LV end-diastolic volume (mL/m^2^)	73.0 ± 17.0	61.2 ± 15.2	<0.001
LV end-systolic volume (mL/m^2^)	25.9 ± 7.6	24.0 ± 6.9	<0.001
LV stroke volume (mL/ m^2^)	47.1 ± 10.4	37.2 ± 8.9	<0.001
LV ejection fraction	64.9 ± 3.9	61.0 ± 3.3	<0.001
Global principal strain (%)	−38.10 ± 3.71	−35.33 ± 3.22	<0.001
Global longitudinal strain (%)	−24.57 ± 2.51	−21.42 ± 2.15	<0.001
Global circumferential strain (%)	−33.35 ± 3.50	−30.90 ± 3.22	<0.001
Global radial strain (%)	47.67 ± 4.27	42.90 ± 3.61	<0.001
Twist (degree)	17.69 ± 7.80	13.34 ± 6.92	<0.001
Torsion (degree/s)	2.04 ± 0.86	1.59 ± 0.80	<0.001

*LV* Left ventricle

**Fig. 3:**
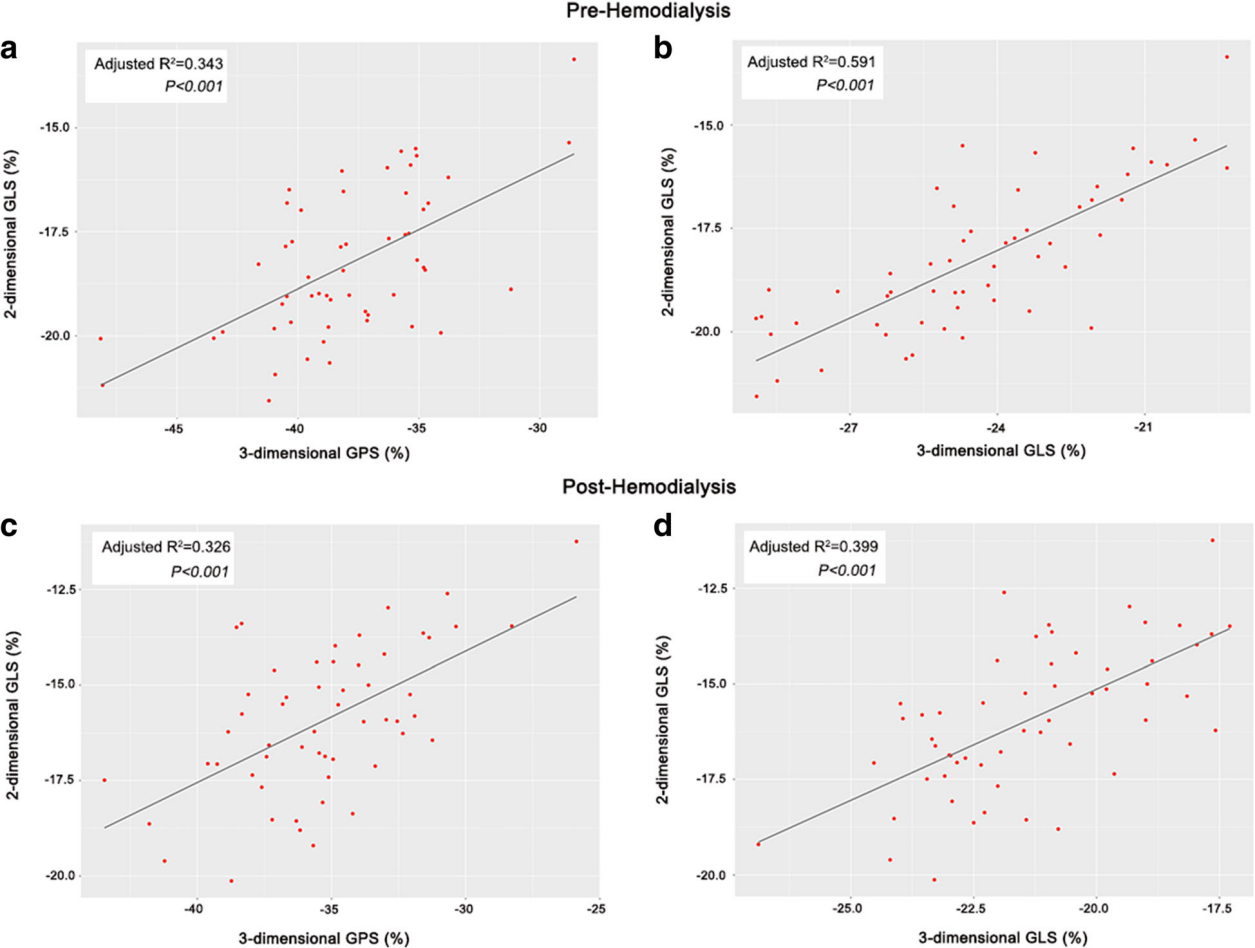
Correlation between 3-dimensional principal and longitudinal strains and 2-dimensional longitudinal strain. Pre-hemodialysis (**a** and **b**); and post-hemodialysis (**c** and **d**). GPS: global principal strain; and GLS: global longitudinal strain

Table 5 compares the volumetric parameters measured by 2D and 3D echocardiography. End-diastolic and end-systolic volumes calculated by 3D echocardiography were significantly larger than those measured by 2D echocardiography. Linear analyses showed that values of 2D global LS were correlated with those of 3D global PS and LS (Fig. 3).

**Table 5:** Differences in left ventricular volumetric parameters measured by 2- and 3-dimensional echocardiography

	3D	2D	*P value*
Pre-HD (n = 58)			
End-diastolic volume (mL/m^2^)	73.0 ± 17.0	67.4 ± 15.7	<0.001
End-systolic volume (mL/m^2^)	25.9 ± 7.6	23.1 ± 6.7	<0.001
Stroke volume (mL/m^2^)	47.1 ± 10.4	43.7 ± 10.5	<0.001
Ejection fraction	64.9 ± 3.9	65.1 ± 4.4	0.770
Post-HD (n = 58)			
End-diastolic volume (mL/m^2^)	61.2 ± 15.2	56.5 ± 17.1	<0.001
End-systolic volume (mL/m^2^)	24.0 ± 6.9	20.8 ± 7.6	<0.001
Stroke volume (mL/m^2^)	37.2 ± 8.9	35.7 ± 10.4	0.029
Ejection fraction	61.0 ± 3.3	63.6 ± 4.3	<0.001

*HD* hemodialysis

**Fig. 4:**
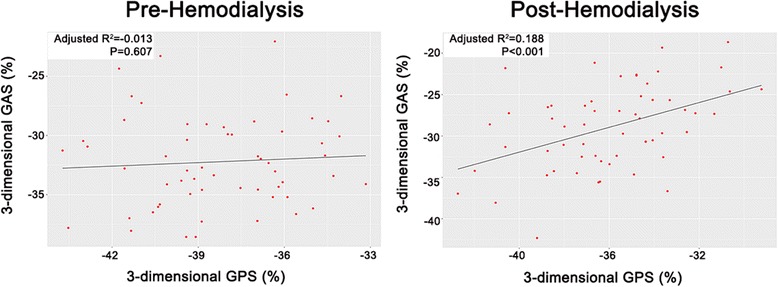
Correlation between 3-dimensional principal and area strains. GPS: global principal strain; and GAS: global area strain

Table 6 shows that global LV strains in all directions are significantly different when analyzed by different analysis software although the same analyzer performed the analysis using the same images, and the correlations were weak or moderate. The correlation between PS and AS was weak in the pre-HD group, but a better correlation was observed in the post-HD group (Fig. 4).

**Table 6:** Comparison of 3-dimensional left ventricular strains using two different software (4D LV analysis: version 3.1, EchoPAC: version 12.0)

	4D LV analysis	EchoPAC	*P* value	ICC	95% CI
Pre-HD (n = 58)					
Global longitudinal strain (%)	−24.57 ± 2.51	−17.10 ± 2.34	<0.001	−0.089	−0.840-0.355
Global circumferential strain (%)	−33.35 ± 3.50	−19.95 ± 2.51	<0.001	0.408	0.000–0.649
Global radial strain (%)	47.67 ± 4.27	53.45 ± 9.64	<0.001	−0.055	−0.783-0.376
Post-HD (n = 58)					
Global longitudinal strain (%)	−21.42 ± 2.15	−15.58 ± 3.21	<0.001	0.116	−0.494-0.477
Global circumferential strain (%)	−30.90 ± 3.22	−17.42 ± 3.71	<0.001	0.570	0.273–0.745
Global radial strain (%)	42.90 ± 3.61	45.79 ± 11.31	0.045	0.304	−0.176-0.588

*ICC* intra-class correlation coefficient, *CI* confidence interval, *HD* hemodialysis

**Fig. 5:**
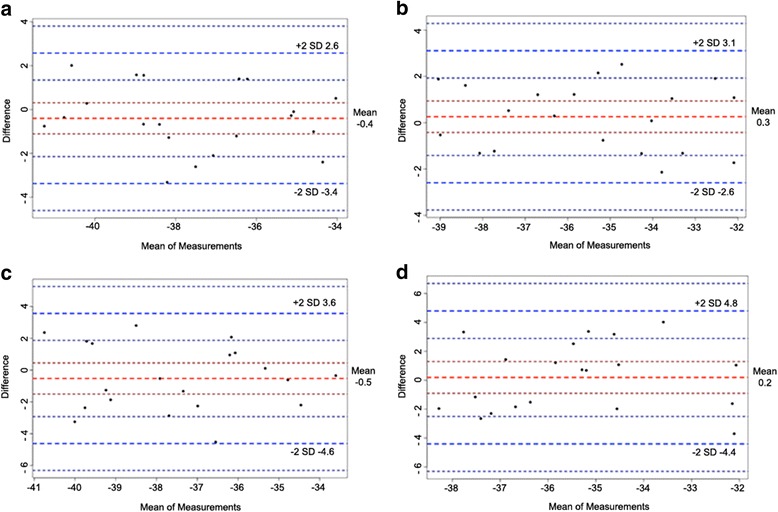
Reliability test for global principal strain of the left ventricle. Bland-Altman analyses for prehemodialysis (**a**: intra-observer; and **b**: inter-observer) and post-hemodialysis (**c**: intra-observer; and **d**: inter-observer) patients

Table 7 summarizes the ICCs of 3D STE strain for intra- and inter-observer measurements. All measurements were in excellent or good agreement, although some inter-observer variations were present, with relatively weak power. Bland-Altman plots for pre- and post-HD PS are presented in Fig. 5.

**Table 7:** Intra-class correlation coefficient (ICC) analysis of intra- and inter-observer variations for strains, twist and torsion of the left ventricle from 3D STE

	Intra-observer variation		Inter-observer variation	
	ICC	95% CI	ICC	95% CI
Pre-HD				
GPS	0.884	0.707–0.954	0.754	0.415–0.899
GLS	0.957	0.891–0.983	0.821	0.547–0.929
GCS	0.815	0.533–0.927	0.628	0.060–0.853
GRS	0.910	0.773–0.964	0.771	0.422–0.910
Twist	0.825	0.557–0.931	0.936	0.838–0.975
Torsion	0.868	0.666–0.948	0.949	0.870–0.980
Post-HD				
GPS	0.898	0.743–0.960	0.619	0.037–0.849
GLS	0.880	0.697–0.952	0.709	0.265–0.885
GCS	0.855	0.633–0.943	0.717	0.285–0.888
GRS	0.923	0.807–0.970	0.814	0.529–0.926
Twist	0.772	0.424–0.910	0.775	0.431–0.911
Torsion	0.713	0.276–0.887	0.746	0.358–0.899

*CI* confidence interval, *3D* three-dimensional, *STE* speckle tracking echocardiography, *HD* hemodialysis, *GPS* global principal strain, *GLS* global longitudinal strain, *GCS* global circumferential strain, *GRS* global radial strain

## Discussion

2D speckle tracking is a useful method for cardiac evaluation. It has been applied in cases of subclinical cardiac dysfunction and for predicting prognosis. 2D echocardiographic images at higher frame rates enable the calculation of strain rate, and thus, it is possible to determine subtle changes in cardiac performance using this technique. However, at least six images should be obtained to measure the global strain and twist values from 2D echocardiography, and one type of strain cannot represent the dynamic cardiac motion. 2D speckle tracking also has limitations in measuring twist because its values are greatly affected by the position of image planes [14].

Real-time 3D echocardiography was first introduced in 1991 [20]. It has advantages for the measurement of LV volume, with a better correlation with cardiac MRI and fewer geometric assumption requirements. Consequently, 3D echocardiography has been suggested as a better option to measure LV volumes in cases of cardiomyopathy or aneurysmal changes that distort the geometry of the left ventricle [3, 4].

Recent advances in cardiovascular imaging techniques have made it possible to measure values of various types of strains from 3D echocardiographic images using the speckle tracking method, which is already widely used and has proven to have clinical importance in 2D imaging [21]. LV 3D strain was reported as a valuable predictor for LV function improvement after myocardial infarction [7] and can be an effective noninvasive method for assessing the twist motion of the left ventricle, as it is less dependent on the position of the image plane [10]. A trial to determine normal reference values for real-time 3-dimensional STE has already been performed [22].

ESRD patients who are regularly undergoing HD have been used as a model for acute preload change in several previous studies. These studies showed that LV and atrial strain values were significantly decreased following preload reduction by HD [23, 24]. 3D echocardiography was also applied on ESRD patients for the evaluation of dynamic LV volume changes during HD [25], and it showed feasibility for clinical application in this group of patients.

PS analysis is a method for describing multidimensional deformations. It identifies the directions along which strain develops and the entity of actual contractions, and therefore, it is particularly well suited for biologic tissues with an underlying structure of muscular fibers along which the stress is generated, such as the heart [12]. In this study, we used vendor-independent software that was previously applied by several investigators [9, 26, 27]. This software was designed to track the real-time 3D and 4D endocardial motion of the left ventricle. It provides the 3D PS value, which is unavailable from 2D images, as well as the traditional longitudinal, circumferential and radial strains.

Three-dimensional PS has proven to be effective in detecting subclinical cardiac abnormalities [26, 27]. AS is also a novel parameter for 3D echocardiographic images, but it only considers the longitudinal and circumferential movement of the left ventricle and does not represent the dynamic 3D motion of the left ventricle or have a correlation with myocardial muscle direction [21]. PS can represent more complex movements of the left ventricle because it considers not only longitudinal and circumferential movement but also twist movement during systole and diastole. It was also shown to correlate with cardiac muscle fiber arrangements. Therefore, it is a useful and novel parameter that can represent complex LV movements during the cardiac cycle.

In this study, twist and torsion calculated using 3D STE were also affected by acute preload changes. LV rotation plays an important role in LV contraction and relaxation. From 2D STE, the difference in the systolic rotation of the myocardium in the apical and basal short-axis planes is referred to as twist and reported in degrees. Data normalized to the distance between the respective image planes are referred to as torsion and reported in degrees/cm [14]. Weiner et al. reported that the rotational movement of the left ventricle measured by 2D STE was affected by preload changes caused by normal saline infusion [28]. However, the measurement of twist using 2D STE requires two apical and basal slices in two different cardiac cycles, and the dependence on the position of two image planes results in a less accurate analysis. The 3D STE used in this study will be a promising tool for further investigations of the rotational movement of the left ventricle.

This study has several strong points compared to other studies investigating changes in the echocardiographic parameters based on preload changes [13, 23, 24, 28].

First, we used newly developed novel strain values for the evaluation of preload reduction. Other strain values, which can be obtained from 2D STE, were also easily and simultaneously calculated during the same cardiac cycle. This benefit made it possible to measure various parameters representing cardiac mechanics more accurately.

Second, the software used was newly introduced vendor-independent software for 3D echocardiographic image analysis. There are several types of software for 3D strain evaluation on the market, but they yield different values in the measurement of 3D strain, even within the same patients [29]. Our results also showed significant vendor dependency even when the same 3D images were analyzed. In the clinic, various types of echocardiographic machines are used for patient care, and 3D echocardiography is already recommended in several guidelines for the evaluation of cardiac function, such as during cancer therapy [30]. This limitation can hamper the application of this technique in the clinical setting. The software that we used in this study was previously used by several investigators and has proven efficacy in 3D echocardiographic image analyses among various types of patients.

Third, 3D strain values could have been acquired from all subjects even though we could not calculate the LS value in 3 subjects due to poor echocardiographic windows. For measurements of acceptable strain values, good-quality real-time 3D echocardiographic images are essential. In addition, a significant learning curve is required even for experienced physicians or echocardiographers.

However, there are also several weak points in this study. The major limitation of this study is that it was performed in a single center with a relatively small number of patients, although the number of individuals enrolled in this study was larger than that in other studies [13, 23, 24, 28].

Second, this study only tracked the endocardium and not the entire myocardium. Compared with the epicardium, endocardial shape change is known to be more associated with global shape change; epicardial, endocardial or global volume; and global rotation and global twist parameters [31].

Third, although the heart rate and frame rate were not significantly different between the pre- and post-HD phases, the relatively higher heart rate post-HD due to acute volume reduction might have affected the frame rate of the 3D echocardiographic images and ultimately led to a greater decrease in all LV mechanical parameters. The frame rates of both groups were greater than 25 frames/s in this study. Yodwut et al. previously demonstrated that frame rates greater than 18 frames/s did not affect the strain values [9]. The lower resolution of the 3D echocardiogram may also influence the quality of the strain analysis and principal strain analysis (PSA) results. We attempted to mitigate this limitation by performing multiple 3D tracking assessments and by validating our data to 2D LS results. Although a previous study reported significant differences in 2D and 3D strain values [32], in this study the values of LS measured by 3D STE were relatively well correlated with both PS and 2D LS. Additionally, 2D STE measurements were not possible in three patients whose 3D STE measurements were available for 3D strain evaluation.

Fourth, HD is not a simple process that leads to only acute volume reduction. During HD, activation of the sympathetic nervous system occurs for the invitation and maintenance of compensatory mechanisms to maintain blood pressure, especially mechanisms involving heart rate and peripheral vasoconstriction [33]. The heart rate was not significantly different after HD, but HD affected blood pressure in this study. The effects of a decrease in blood pressure on the echocardiographic parameters could not be excluded.

Fifth, a study that aimed to determine normal reference values of 3D echocardiographic strain showed that there were differences in normal values between different segments, walls and levels of the left ventricle. There is still no accepted reference value of 3D strain, and there are significant inter-vendor differences in the measured values [29]. We used vendor-independent software in this study and compared only global strain values before and after acute preload reduction in the same subjects; therefore, this limitation could be overcome in this study.

## Conclusion

This study showed that deformation parameters measured from 3D echocardiographic images using the speckle tracking method are affected by acute preload changes. 3D echocardiography can be used to calculate strain, twist and torsion, which can represent complex LV mechanics, from a single image. However, all parameters representing LV systolic function, including the novel parameter of PS, were affected by acute preload changes. Therefore, the values of strain, twist and torsion acquired from 3D STE should be interpreted with caution and with consideration of the preload status of the patient. The findings of this study are important for patients in critical settings, such as acute heart failure and shock patients, who are experiencing significant volume shifts due to the disease and the treatment. More studies are needed to explore the prognostic value of PS, a novel parameter that reflects actual deformation along the principal direction, especially in ESRD patients who are very susceptible to cardiovascular complications.
